# The Treatment of Rickets

**Published:** 1893-02-25

**Authors:** 


### PADDINGTON GREEN CHILDREN'S HOSPITAL.

### The Treatment of Rickets.

The treatment of rickets forms a large part of the work at a children's hospital. At Paddington Green the milder cases are treated as out-patients, while the more severe and complicated cases are admitted to the wards. In this paper we shall combine the instructions usually given to mothers for the home management of patients, with the more special treatment carried out in the hospital.

I.—Home Surroundings, Dress, and Exercise.

As fresh air is most essential for a rickety child, the home ought to allow of thorough ventilation. Hence rooms situated in an area sunk below the level of the street, or in a house built up closely at the back, or in a narrow street, are to be avoided. The nursery and bedroom ought to be at the top of the house, and the windows should be large, and capable of being opened. The ventilation commonly trusted to is that caused by the current of air between the fire and the door, but in addition it is advisable to have a solid block of wood, about two and a half inches deep, placed underneath the lower window sash, which will allow of a constant supply of fresh air. This should be used day and night during the whole of the summer and the greater part of the winter. In the bedroom a small fire should be kept burning all night during very cold weather. In hospital the patient sleeps alone in a cot, which is placed in a part of the ward away from draughts. The sides of the cot are open, and there is no curtain over the top, so as to allow of a free play of air. The bed-clothes are warm and light, and the patient sleeps between blankets. To check the profuse sweating about the head, a small, firm pillow stuffed with horsehair is used, on which the head rests without sinking deeply in. The effect of soft pillows in rickety cases is that in a short time after the child falls asleep his head is surrounded by a hot, wet cushion, and this condition is accompanied with extreme discomfort and liability to catch cold.

To avoid a chill from exposure on kicking off the clothes at night, a common act in rickety children, the patient wears a long flannel nightdress, which is fastened round the neck, and wrists, and below the feet. By this means he is prevented from exposing his body or limbs, however restless he may be. The bed clothes and nightdress are carefully aired after use, especially in those cases in which sweating is profuse. The essential part of the clothing by day consists in a complete covering of flannel, and the following are the garments used: (1) A flannel vest, fitting closely round the neck, and loosely elsewhere. All the outer clothing of the chest must also be loose, so as to allow of free expansion of the lungs. (2) A well-fitting flannel binder round the abdomen, extending from just below the hips to the lowest part of the sternum. To this shoulder straps are attached, so as to prevent it from slipping down. (3) Flannel drawers extending to the knees and buttoned above to the binder. (4) Warm stockings extending above the knees and fastened to the drawers. Woollen gaiters are worn above these in cold weather, and should extend well up the thighs. The modifications in the above necessary to adapt them to the age of the child may be left to the mother.

The child is washed all over night and morning with soap and warm water. If there is a tendency to skin eruptions, the soap is dispensed with, or one of the superfatted soaps is used. The temperature of the bath for a rachitic infant is 90 degrees, and after twelve months it may be reduced to 80 degrees, and even lower if the child stands it well. The colder the bath, the more beneficial it is to the patient, provided that no depression or feeling of cold follows. The skin is dried with a rough warm towel, special attention being paid to the head and ears. The limbs are then thoroughly rubbed with the open hand until the skin is reddened. Each limb is taken separately, the rest of the body being kept carefully covered, and the rubbing is directed from the extremity upwards. This is a great preventive of the cold hands and feet to which these patients are so subject.

Rickety children ought to be out of doors most of the day in fine weather. They should be carried as little as possible, and never by children only slightly older than themselves. A perambulator, in which they can lie at full length, is the best means of locomotion, and in this a hot bottle may be placed with advantage in very cold weather.

II.—Food and Feeding.

If rickets develops in a child entirely breast-fed, and under seven months of age, it is plain that the nourishment is insufficient, and cow's milk must be given once or twice a day in addition to the breast. If the child is over seven months, it is better to stop the nursing entirely. In hand-fed babies the presence of rickets is almost invariably associated with the abuse of artificial foods, and a deficient supply of fresh cow's milk. A diet is therefore ordered consisting of from one to two pints of cow's milk in the twenty-four hours, suitably diluted with barley water. A pinch of salt is added to each bottle of milk, and instead of sweetening the milk with sugar, which is frequently done to such an extent as to produce fermentative disorders in the stomach, half a teaspoonful of the fluid extract of malt is used. The mixture of milk and malt is very palatable. After nine months some extra nourishment may be added, in very small quantity at first, and the motions must be carefully observed in order to see that the food has been thoroughly digested and absorbed. In the hospital a patient of twelve months is allowed the following extra dietary: Milk and egg, gravy and bread crumb, well-mashed potato with milk and salt, gruel and oatmeal porridge, stewed fruits, and rice, sago, or tapioca puddings, made with milk. At eighteen months a little fresh fish may be added. As a prominent factor in the production of rickets is the absence of fatty materials from the food, the addition of cream (half an ounce thrice daily), or, in the case of older children, fat bacon is extremely beneficial. The regularity of the meals must also be attended to. For a child from nine to fifteen months, the rule is to feed every three hours by day and once during the night, namely, between ten p.m. and six a.m.; and after that age, to feed every four hours by day and not at all during the night. The question of diet has already been fully discussed in previous papers,* and need not be further referred to here.

*(To be continued.)*

* The Hospital, November 12th and 19th, 1892.

